# Mild Cognitive Impairment Subtypes and Type 2 Diabetes in Elderly Subjects

**DOI:** 10.3390/jcm9072055

**Published:** 2020-06-30

**Authors:** Silvia Valenza, Lucia Paciaroni, Susy Paolini, Anna Rita Bonfigli, Mirko Di Rosa, Rosa Anna Rabini, Elena Tortato, Paolo Pelliccioni, Giuseppe Pelliccioni

**Affiliations:** 1 Neurology Department, Italian National Research Center on Aging (IRCCS INRCA), 60100 Ancona, Italy; silvia.valenza@ospedalimarchenord.it (S.V.); l.paciaroni@inrca.it (L.P.); s.paolini@inrca.it (S.P.); 2 Psychology Department, Azienda Ospedaliera Ospedali Riuniti Marche Nord, 61121 Pesaro-Fano, Italy; 3 Scientific Direction, Italian National Research Center on Aging (IRCCS INRCA), 60100 Ancona, Italy; a.bonfigli@inrca.it; 4 Unit of Geriatric Pharmacoepidemiology and Biostatistics, Italian National Research Center on Aging (IRCCS INRCA), 60124 Ancona, Italy; m.dirosa@inrca.it; 5 Metabolic Diseases and Diabetology Department, Italian National Research Center on Aging (IRCCS INRCA), 60100 Ancona, Italy; rosaanna.rabini@sanita.marche.it (R.A.R.); e.tortato@inrca.it (E.T.); 6 Eye Clinic, Polytechnic University of Marche, 60126 Ancona, Italy; paopel@hotmail.it

**Keywords:** cognitive functions, mild cognitive impairment, dementia, aging, type 2 diabetes, neuropsychological test, Alzheimer’s disease

## Abstract

Background: Type 2 diabetes (T2D) is correlated to amnestic mild cognitive impairment (aMCI) and to non-amnestic mild cognitive impairment (naMCI). This study evaluated whether the T2D variable characterizes a peculiar cognitive profile in elderly patients. Moreover, it explores the association between glycated hemoglobin levels (HbA1c), T2D duration, insulin and oral hypoglycemic agent treatment, and cognition in elderly diabetic patients. Methods: Detailed neuropsychological battery was used to diagnose MCI subtypes. A total of 39 MCI subjects with T2D (T2D-MCI) and 37 MCI subjects without T2D (ND-MCI), matched for age, educational level, and Mini-Mental State Examination score, were included. Results: ND-MCI performed worse in memory and language domains than T2D-MCI. The amnestic subtype is more frequent among ND-MCI and non-amnestic subtype in T2D-MCI. In T2D-MCI, high HbA1c levels correlate with episodic memory (immediate recall) and T2D duration. Some indexes of episodic memory (immediate recall), attention, and visual-spatial ability correlate with insulin treatment. Conclusions: An association between T2D and non-amnestic MCI is suggested. In the T2D-MCI group, significant associations between insulin treatment and memory (immediate recall), complex figure copy, and attention were found.

## 1. Introduction

Type 2 diabetes (T2D), a complex metabolic disease that may have significant effects on various organs of the body, has been found to be a risk factor for the development of dementia in multiple studies [1,2,3]. The mechanisms linking T2D with neurodegenerative disease, in particular with Alzheimer’s disease (AD), remain unclear [4], but may include both cerebrovascular and neurodegenerative processes. Vascular risk factors associated with T2D, including hypertension, dyslipidemia, and obesity, have all been linked to a higher risk of dementia [5], while with regard to the neurodegenerative mechanisms, it has been observed that T2D can adversely affect the accumulation and processing of the amyloid beta protein [6,7]. Moreover, a long T2D duration may be associated with greater cerebral macrovascular disease, as well as clinical and subclinical cerebral infarctions that may impair cognitive function. Insulin has proved to be a crucial element for neurological functions. T2D and chronic peripheral hyperinsulinemia are associated with impairment in memory and cognitive functions. Furthermore, one important common mechanism between T2D and AD is impaired insulin signaling; moreover, a form of toxic amyloid can damage neuronal insulin receptors and affect insulin signaling and cell survival [8]. Recurrent or chronic hypoglycemia caused by insulin treatment may also contribute to permanent cognitive impairment [9].

Various studies have found an association in elderly patients between T2D and mild cognitive impairment (MCI) [10,11], a condition manifesting in initial cognitive deficits that do not significantly interfere with the autonomy or social behavior of the patient [12]. MCI incidence is higher in individuals with T2D than in those without T2D [13]. T2D is considered a risk factor for MCI and this risk may be associated with the duration of T2D, use of glucose-lowering medications, and the degree of glucose control [14]. Moreover, Ma et al. showed that the presence of MCI in the T2D accelerated the median progression to dementia by 2.74 years [15].

Depending on whether memory is affected by the deterioration or not, MCI can be defined as amnestic MCI (aMCI) in which case the cognitive amnestic domain is primarily involved, and as non-amnestic MCI (naMCI) in which case the memory of the subject is not impaired.

However, research results on the association between T2D and the MCI subtypes are not entirely consistent, with some studies indicating greater memory impairment [16], others showing a cognitive profile characterized by a deficit in executive-attention and further functions without amnestic alteration [17].

This inconsistency in findings may largely be due to differences in the design of the studies or the criteria for the diagnosis of T2D and MCI. 

Our study utilized an extensive neuropsychological battery to compare the cognitive performance of T2D-MCI and MCI subjects without diabetes (ND-MCI) and to determine whether the T2D variable characterizes a peculiar cognitive profile in elderly subjects. In addition, we aimed to explore the association between the levels of glycated hemoglobin (HbA1c), the duration of T2D, treatment with insulin, oral hypoglycemic agents, and cognition in T2D-MCI.

## 2. Methods

### 2.1. Study Population and Recruitment

The study was retrospective, observational, and cross-sectional with the recruitment site located at the INRCA Research Hospital in Ancona, Italy. A total of 39 T2D-MCI patients were compared with 37 ND-MCI matched patients. Both groups of patients visited the Neurology Department due to worries about their cognitive state. 

Inclusion criteria allowed for subjects between the age of 70 and 85 with ≥5 years of formal education, scoring ≥24 on the global cognitive functioning test (Mini-Mental State Examination, MMSE) [18], demonstrating autonomy in everyday life, and having preserved or only slightly impaired functioning in complex daily activities. Autonomy in basic functions was investigated through the questionnaire Activities of Daily Living (ADL) [19], and that of complex functioning through Instrumental Activities of Daily Living (IADL) [20]. In addition to this, HbA1c, Body Mass Index (BMI), presence of hypertension, neuropathy, nephropathy, chronic renal failure, retinopathy, supra-aortic trunks arterial disease, obliterative arteriopathy of the lower limbs, and myocardial ischemia were clinically assessed according to the criteria of the Standards of Medical Care in Diabetes [21]. In particular, nephropathy was diagnosed on the basis of a urine albumin/creatinine ratio >30 mg/g, while chronic renal failure was present if the glomerular filtration rate was <60 mL/min/1.73 m^2^.

The exclusion criteria consisted of psychiatric and neurological disorders (psychosis and major depressive disorders, cerebrovascular diseases, head injuries, cerebral tumors, Parkinson’s disease, epilepsy, dementia). During the recruitment, the presence of T2D was assessed by clinical history and through laboratory assays, according to the diagnostic criteria established by the American Diabetes Association [21].

Patients with a new diagnosis of diabetes were treated according to clinical practice at the Diabetology Department of our institute. 

The mean duration of diabetes was 17.5 ± 11.9 years. Seventeen subjects were treated with oral antidiabetic drugs, 14 with insulin of which 8 were treated with a combined therapy (insulin + oral antidiabetic agents), and only 8 subjects with diet therapy. In particular, among subjects treated with insulin, 10 subjects used rapid and long-acting insulin; one subject used rapid, long-acting, and pre-mixed insulin; one subject used only rapid-acting insulin; and 2 subjects used only long-acting insulin. Overall, the duration of insulin treatment was 8.6 ± 6.4 years.

The two groups (T2D-MCI and ND-MCI) were matched for age, educational level, and MMSE score. Information collected included data on vital signs, anthropometric factors, medical history, and behavior.

All participants gave their written informed consent for the enrollment in the present study. The study was approved by the INRCA IRCSS Ethics Committee of Ancona, Italy (Institutional Review Board), and the study protocol conformed to the principles of the Declaration of Helsinki.

The data sets supporting the results of this article are available in the Neurology Department INRCA IRCSS Ancona, Italy, repository.

### 2.2. MCI Diagnostic Criteria

Diagnosis of MCI was made according to Petersen’s criteria [12]: (1) subjective cognitive complaint, preferably confirmed by an informant; (2) objective impairment in performance on the cognitive test in the assessment battery, at least greater than 1.5 standard deviation below the scores of age- and education-matched normal aged individuals; (3) preserved global cognitive function; (4) essentially normal functional activities; (5) absence of dementia.

Mild cognitive impairment is subclassified into four subtypes by the presence of impairment in the memory domain (amnestic vs non-amnestic) and the number of impaired cognitive domains (single vs multiple): aMCI single-domain (aMCI-sd), aMCI multi-domain (aMCI-md), naMCI single-domain (naMCI-sd), naMCI multi-domain (naMCI-md) [22].

Given the number of subjects available, we considered it appropriate to classify patients on the basis of the presence or absence of the memory impairment, dividing them between aMCI and naMCI.

Each subject received a clinical evaluation and a standardized neuropsychological battery to verify the presence of the inclusion criteria. Neuropsychological assessment (Table 1) provided a large number of tests that allowed for the quantification of the subjects’ performance in memory and other cognitive tests (language, visual-spatial skills, attention, and executive functions) and classification of the various subtypes.

To verify essentially normal functional activities, informant information was collected in all cases.

According to the pattern of impairment on the neuropsychological evaluation, all patients with MCI were classified as having aMCI if they had memory deficits in at least 2 memory tests. The patients were classified as having naMCI if they had at least 2 tests impaired in one non-memory domain [23]. In case of patients failing in at least 2 memory tests and having at least 2 tests impaired in one non-memory domain, they were classified only as aMCI.

### 2.3. Statistical Analysis

Firstly, the sample was analyzed for characteristics in terms of age, gender, years of education, cognitive state (identified by the MMSE score), HbA1c, Body Mass Index (BMI), presence of hypertension, neuropathy, nephropathy, chronic renal failure, retinopathy, supra-aortic trunks arterial disease, obliterative arteriopathy of the lower limbs, and myocardial ischemia comparing T2D-MCI and ND-MCI. For all continuous variables, the Shapiro–Wilk test for normality was calculated. Apart from gender, for which the proportions were analyzed, we also calculated mean and standard deviation for normally distributed variables, and the median and interquartile range for non-normally distributed variables. The same evaluation was carried out in terms of neuropsychological tests, reporting means and standard deviations (or medians and interquartile ranges) according to the two groups of subjects. To test if the differences between T2D-MCI and ND-MCI were statistically significant, parametric (Student-*t*) and non-parametric (Mann–Whitney U) tests were performed as appropriate. The association between T2D and neuropsychological tests was further investigated with stepwise linear regression analysis including the following confounders: gender, age, education, BMI, hypertension, neuropathy, nephropathy, chronic renal failure, retinopathy, supra-aortic trunks arterial disease, obliterative arteriopathy of the lower limbs, and myocardial ischemia. For normally distributed outcome variables, ordinary least squares linear regressions were estimated, while for non-normally distributed dependent variables, quantile (median) linear regressions were estimated. Thereafter, the chi-squared test was performed to evaluate the differences between MCI subtypes in T2D-MCI and ND-MCI subjects. Pearson’s correlation coefficients for normally distributed variables and Spearman’s rank correlation coefficients for non-normally distributed variables were calculated in order to evaluate the relationship among the neuropsychological tests and clinical features for T2D-MCI subjects.

All analyses were performed using SPSS 17.0 (SPSS Inc., Chicago, IL, USA) and the STATA version 15.1 Statistical Software Package for Windows (StataCorp, Collge Station, TX, USA); a value of 0.05 was considered significant.

## 3. Results

The demographic variables and clinical characteristics of T2D-MCI and ND-MCI are shown in Table 2. All continuous variables are normally distributed with the exception of the MMSE (*p* = 0.59). There were no significant differences of gender, age, education level, and MMSE score between the two groups.

Table 3 shows the significant differences between T2D-MCI and ND-MCI in neuropsychological tests. ND-MCI showed a significantly worse performance in some indexes of memory compared to T2D-MCI (*p* ≤ 0.05). Moreover, the comparison between the two groups revealed significant differences in the language tests: ND-MCI subjects obtained statistically significant lower scores in Noun Naming and Semantic Fluency tests (fluency for semantic categories and animal fluency) (*p* ≤ 0.05).

These results are also confirmed after the introduction of possible confounders in the multivariate analysis (Table 4).

Evaluating confounders, age, education, BMI, and clinical complications of diabetes showed a statistically significant association with the performance on neuropsychological tests.

**Figure 1:**
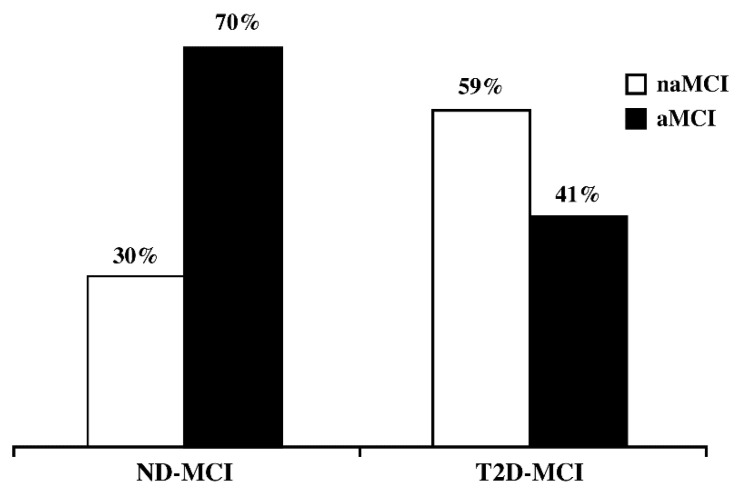
Prevalence of MCI subtypes in ND-MCI and T2D-MCI (%). *p* from χ^2^ test =0.010.

Figure 1 shows the prevalence of MCI subtypes (amnestic or non-amnestic) according to the presence or absence of T2D. It emerges that in the T2D-MCI group, naMCI subjects are more frequent (59% naMCI vs 41% aMCI), while in the ND-MCI group aMCI are prevalent (30% naMCI vs 70% aMCI). This result was tested by means of the chi-square test and the differences are statistically significant (*p* = 0.010).

In a secondary analysis, we also examined the correlation between HbA1c levels, insulin treatment, oral hypoglycemic agents, T2D duration, and cognition in T2D-MCI (Table 5).

A significant inverse association was found between HbA1c and episodic memory (immediate recall). In addition, further significant inverse correlations were found between the duration of T2D and visual spatial memory (immediate recall), complex figure reproduction, and Multiple Features Target Cancellation Test (MFTC) (accuracy index). Significant inverse associations between insulin treatment and memory (immediate recall), complex figure copy, and attention were also seen, as well as a significant direct association between insulin treatment and MFTC Test (time index).

**Figure 2:**
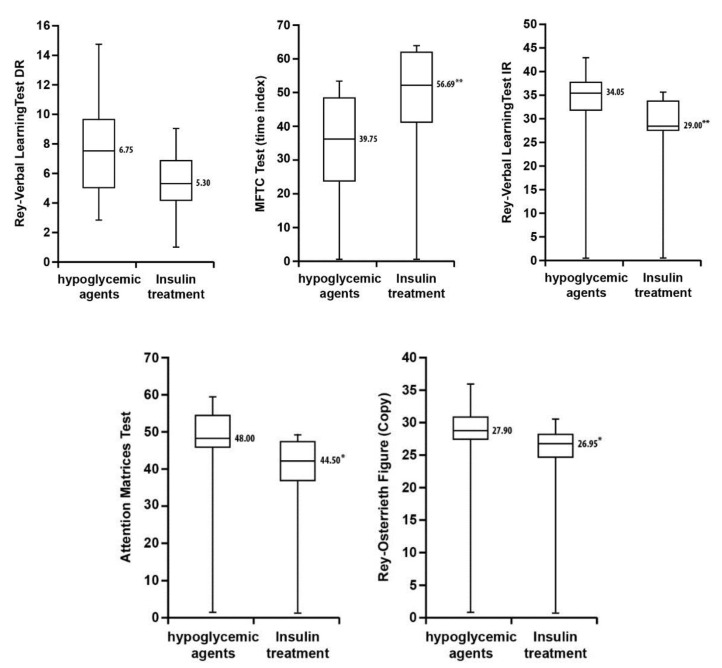
Box-and-whisker plot showing differences in cognitive function tests for T2D-MCI on insulin therapy and hypoglycemic agents. Data are presented as median (interquartile range). Statistical significance tested with Mann–Whitney U tests (* *p* < 0.05; ** *p* < 0.01).

A bivariate analysis between oral hypoglycemic agents and cognitive function did not reveal any significant results (Figure 2).

## 4. Discussion

Increasing evidence indicates a potential effect of T2D on the risk of cognitive dysfunction; however, the correlation between T2D and a cognitive impairment risk is still unclear [38].

Our study suggests that T2D is associated with an increased risk of a subtype of cognitive impairment. In fact, we found that among T2D-MCI the naMCI subtype prevails, while aMCI is most frequently found in ND-MCI.

Differences in the association of diabetes across MCI subtypes raise questions regarding the role of diabetes in the etiology and prognosis of MCI subtypes. Some studies in fact have found a relationship between diabetes mellitus type 2 and MCI [39,40], an intermediate stage between the expected cognitive decline of normal aging and the more serious decline of dementia.

In accordance with the results of a wide variety of highly reproducible studies, aMCI generally characterizes a subtype of patients who have a greater likelihood of progressing to AD [41], while naMCI seems to be related to non-AD forms [42].

In our patients, the T2D-MCI group showed less damage to memory functions compared to those without T2D (ND-MCI). These data were also corroborated by the significantly worse scores of the ND-MCI group in two indices of the memory test Free and Cued Selective Reminding Test (FCSRT) (Immediate Total Recall (ITR) and Index of Sensitivity Cueing (ISC)). 

FCSRT can be used to assess specific features of episodic memory impairment as it controls for encoding at the time of study and provides retrieval cues at the time of memory testing. Furthermore, ITR and ISC indices, which control for the encoding and the magnitude of sensitivity to semantic cues, are proposed as specific markers of a deficit in hippocampal memory [43].

In T2D-MCI, the memory deficit is primarily related to a reduced attention capacity and inefficient strategies for information recovery, resulting from the dysfunctional executive systems and, therefore, non-amnestic in nature. 

On the other hand, in ND-MCI, the memory deficit is both deeper and connected to problems of storage and authentic encoding, therefore, amnestic in nature.

Our results are confirmed also after the introduction in the multivariate analysis of other factors associated with T2D/ND and MCI (gender, age, education, BMI, hypertension, neuropathy, nephropathy, chronic renal failure, retinopathy, supra-aortic trunks arterial disease, obliterative arteriopathy of the lower limbs, and myocardial ischemia). 

In addition, ND-MCI shows greater difficulties in the lexical access, with a semantic memory damage compatible with the AD pattern [44]. This agrees with some studies that have not only shown a different pattern between T2D-MCI and ND-MCI, but also a different trend over time: while T2D-MCI usually remains stable, ND-MCI tends to get worse especially in the amnesic components [45,46]. However, different neuropathological studies showed inconclusive results, not finding an increased frequency of any particular type of dementia in diabetic patients submitted to autopsy [47]. 

The correlations between clinical variables and cognitive tests provided further confirmation of these data. In our study, the relationship between the level of glycated hemoglobin and the cognitive tests was also assessed. The resulting data show that an increase of glycated hemoglobin is associated with a significant reduction in the scores on a test on verbal memory for immediate recall, and that the duration of T2D is associated with worse attention functions and visual-spatial ability. We furthermore found that the use of insulin inversely correlated to memory and visual-spatial tests, highlighting that patients with insulin therapy are prone to neuronal damage and cognitive impairment [48]. Other prospective studies evaluated the effects of antidiabetic therapy on conversion from T2D-MCI to dementia. Ma et al. found that dementia risk was significantly decreased among patients using oral hypoglycemic agents compared to insulin-treated patients [15]. These findings are confirmed by different studies, which have found that T2D increases the risk of dementia, principally in patients treated with insulin [2,5,49]. 

The nature of this association seems to be insulin resistance (leading cause of T2D) in brain cells, with an impairment in their activities, thereby determining the onset of typical symptoms of AD [49,50].

In addition, animal studies have demonstrated that neurons can generate insulin, and insulin receptors have been found in key areas of the brain involved with AD pathology and cognitive function [51,52]. Due to the role of insulin in glucose regulation, insulin abnormalities may likely impair cerebral glucose metabolism, a characteristic which has been demonstrated in both AD and MCI brains [53,54]. 

The molecular mechanisms underlying this crosstalk are still elusive, as well as how central and peripheral insulin signaling operate in AD [55].

Further studies of large cohorts of diabetic patients would be necessary to examine the effects of drug dosage and adherence, which may have greater impacts on patients who take insulin than on patients who take oral agents.

In our study, we found no correlation between oral treatment for T2D and cognitive tests.

Our findings therefore provide further evidence that the severity of T2D is associated with a greater compromise of the memory, and that milder forms of T2D may be related with an attention-executive functioning deficit.

The principal strength of the present study is the detailed neuropsychological examination, and the procedure we followed has ensured that the group of selected subjects was characteristically homogeneous from a neuropsychological point of view to ensure a suitable specimen observation. 

The main limitations of our study are the small size of our sample and the fact that the data used were cross-sectional, which precludes the determination of causation and monitoring of changes over time. Moreover, different patients of our study refused cerebrospinal fluid evaluation, and also amyloid and fluorodeoxyglucose-PET were performed only in few patients.

Studies with larger numbers of patients should be performed to confirm our preliminary results, and findings regarding insulin use need further support in bigger samples. Future research in following up these subjects in an ongoing longitudinal cognitive aging project may offer further insights into the inter-relationships of T2D, HbA1c, and cardiovascular risk factors with dementia and aging. 

## 5. Conclusions

In conclusion, our study suggests an association between T2D and naMCI. In addition, our findings showed that in the T2D-MCI group, patients treated with insulin have worse cognitive performances with a deficit spreading to several cognitive domains, such as memory, attention, executive functions, and visual-spatial abilities.

MCI early identification and strict monitoring in T2D patients may be relevant to delay the progress of cognitive decline, and the choice of hypoglycemic treatment should be carefully defined.

## Figures and Tables

**Table 1:** Neuropsychological battery.

Attention and executive functions	Attention Matrices Test [24]
	Trail Making Test A, TMT-A, and Trail Making Test, TMT-B [25]
	Stroop Test [26]
	Weigl’s Sorting Test [27]
	Multiple Features Target Cancellation Test (MFTC): time; accuracy; error [28]
	Frontal Assessment Battery (FAB) [29]
	Phonemic Fluency (FAS) [30]
Memory	Digit span [31]
	Rey Auditory Verbal Learning Test: immediate and delayed recall [32]
	Prose Memory Test [33]
	Free and Cued Selective Reminding Test (FCSRT): Immediate Free Recall (IFR); Immediate Total Recall (ITR); Delayed Free Recall (DFR); Delayed Total Recall (DTR); Index of Sensitivity Cueing (ISC) [34]
	Rey–Osterrieth Complex Figure B: immediate and delayed recall [35]
Language	Animal fluency [36]
	Fluency for semantic categories [37]
	Oral comprehension [36]
	Verbal Naming [36]
	Noun Naming (CAGI) [37]
Visual constructional ability	Rey–Osterrieth Complex Figure B copy [35]

**Table 2:** Demographic data and clinical characteristics of mild cognitive impairment (MCI) with type 2 diabetes (T2D)-MCI and MCI subjects without diabetes (ND-MCI).

	T2D-MCI *n* = 39	ND–MCI *n* = 37	*p*
Age *, mean (sd)	74.21 (4.58)	76.68 (6.30)	0.06
Female, *n* (%)	18 (46.15)	22 (59.46)	0.25
Education (years) *, mean (sd)	7.44 (2.98)	8.27 (3.90)	0.30
MMSE ^, median (iqr)	26.30 (2.40)	25.70 (2.00)	0.10
HbA1c *, mean (sd)	7.62 (1.67)	5.30 (0.80)	0.01
BMI *, mean (sd)	28.33 (5.23)	23.63 (2.50)	0.00
Hypertension, *n* (%)	26 (66.67)	18 (48.65)	0.11
Neuropathy, *n* (%)	6 (15.38)	0 (0.00)	0.01
Nephropathy, *n* (%)	3 (7.69)	0 (0.00)	0.09
Chronic Renal Failure, *n* (%)	2 (5.13)	1 (2.70)	0.59
Retinopathy, *n* (%)	13 (33.33)	0 (0.00)	0.00
Supra-aortic Trunks Arterial Disease, *n* (%)	2 (5.13)	1 (2.70)	0.59
Obliterative Arteriopathy of Lower Limbs, *n* (%)	5 (12.82)	2 (5.41)	0.26
Myocardial Ischemia, *n* (%)	9 (23.08)	14 (37.84)	0.16

*p* from *t*-test for normally distributed variables (*), Mann–Whitney U test for non-normally distributed variables (^), and χ² for dichotomic variables.

**Table 3:** Significative comparison of performance on neuropsychological tests between T2D-MCI and ND-MCI.

	T2D-MCI		ND-MCI		*p*
	Mean/Median	SD/IQR	Mean/Median	SD/IQR	
FCSRT ITR (immediate total recall) *	35.25	1.31	34.38	2.25	0.04
FCSRT DTR (Delayed Total Recall) *	11.60	0.75	11.03	1.58	0.05
FCSRT ISC (Index of Sensitivity of Cueing) *	0.95	0.06	0.91	0.11	0.03
Rey Auditory Verbal Learning Test (delayed recall) ^	6.90	3.70	5.30	4.70	0.02
Prose Memory Test ^	12.50	7.50	10.00	4.50	0.01
Rey–Osterrieth Complex Figure B (delayed recall) ^	16.51	5.59	13.98	5.27	0.05
Fluency for semantic categories ^	40.30	9.19	36.30	8.42	0.01
Animal fluency ^	16.50	6.60	14.00	4.40	0.03
Noun Naming (CAGI) *	45.04	2.03	44.00	1.95	0.02

Normally distributed variables (*) are expressed as mean, standard deviation (SD) and *p* from *t*-test; non-normally distributed variables (^) are expressed as median, interquartile range (IQR), and *p* from Mann–Whitney U test.

**Table 4:** Effect of T2D on each neuropsychological test in fully adjusted stepwise linear regression analysis: un-standardized betas (B) ± standard error (SE) and level of significance (*p*).

Independent Variables:	B ± SE	*p*
FCSRT ITR (immediate total recall) *	1.09 ± 0.41	0.01
FCSRT DTR (Delayed Total Recall) *	0.70 ± 0.26	0.01
FCSRT ISC (Index of Sensitivity of Cueing) *	0.06 ± 0.02	0.01
Prose Memory Test ^	3.00 ± 0.63	0.00
Rey–Osterrieth Complex Figure B (delayed recall) ^	5.49 ± 1.32	0.00
Fluency for semantic categories ^	3.99 ± 1.32	0.00
Animal fluency ^	2.75 ± 1.03	0.01
Noun Naming (CAGI) *	1.29 ± 0.48	0.01

Coefficients were estimated with ordinary least squares linear regression for normally distributed variables (*) and with quantile (median) linear regression for non-normally distributed variables (^). Each model is adjusted for gender, age, education, BMI, and clinical complications of diabetes (hypertension, neuropathy, nephropathy, chronic renal failure, retinopathy, supra-aortic trunks arterial disease, obliterative arteriopathy of the lower limbs, and myocardial ischemia).

**Table 5:** Pearson’s and Spearman’s correlations (r) between cognition and clinical features in T2D-MCI.

	HbA1c	Duration of T2D	Insulin Treatment
Rey Auditory Verbal Learning Test (immediate recall) *	r = −0.37 *p* = 0.03	r = −0.27 *p* = 0.11	r = −0.39 *p* = 0.02
Rey Auditory Verbal Learning Test (delayed recall) ^	r = −0.13 *p* = 0.47	r = −0.02 *p* = 0.89	r = −0.33 *p* = 0.06
Rey–Osterrieth Complex Figure B (immediate recall) ^	r = −0.30 *p* = 0.09	r = −0.38 *p* = 0.02	r = −0.25 *p* = 0.14
Rey–Osterrieth Complex Figure B (copy) ^	r = −0.28 *p* = 0.11	r = −0.45 *p* = 0.01	r = −0.33 *p* = 0.05
Attention Matrices Test ^	r = −0.32 *p* = 0.58	r = −0.24 *p* = 0.16	r = −0.45 *p* = 0.01
MFTC Test (time index) *	r = 0.21 *p* = 0.23	r = 0.21 *p* = 0.22	r = 0.37 *p* = 0.02
MFTC Test (accuracy index) *	r = −0.18 *p* = 0.31	r = −0.34 *p* = 0.03	r = 0.09 *p* = 0.34

r = Pearson’s correlation coefficients for normally distributed variables (*) or Spearman’s correlation coefficients for non-normally distributed variables (^).

## Abbreviations

ADL	Activities of Daily Living;
AD	Alzheimer’s disease;
aMCI	amnestic mild cognitive impairment;
aMCI-md	amnestic mild cognitive impairment, multi-domain;
aMCI-sd	amnestic mild cognitive impairment, single-domain;
FCSRT	Free and Cued Selective Reminding Test;
FCSRT ITR	Free and Cued Selective Reminding Test, Immediate Total Recall;
FCSRT ISC	Free and Cued Selective Reminding Test, Index of Sensitivity of Cueing;
HbA1c	glycated hemoglobin levels;
IADL	Instrumental Activities of Daily Living;
MCI	Mild Cognitive Impairment;
MMSE	Mini-Mental State Examination;
naMCI	non-amnestic mild cognitive impairment;
naMCI-md	non-amnestic mild cognitive impairment, multi-domain;
naMCI-sd	non-amnestic mild cognitive impairment, single-domain;
ND-MCI	MCI subjects without diabetes;
T2D	type 2 diabetes;
T2D-MCI	MCI subjects with type 2 diabetes.
